# Supplementary material for: Effectiveness of food supplement on treatment outcomes and quality of life in pulmonary tuberculosis: Phased implementation approach
Source: PLoS One. 2024 Jul 16;19(7):e0305855. doi: 10.1371/journal.pone.0305855 (PMC11251611; doi:10.1371/journal.pone.0305855)
Supplement: Attachment — Submitted filename: PONE-D-23-12272 R3 Response to reviewers 12022024.docx [file pone.0305855.s005.docx]

**PONE-D-23-12272 - Response to Reviewers comments (R3)**

Reviewer #2: The topic of providing a nutritional intervention for persons with TB is important, and the results present compelling evidence for the benefits of such an intervention. However, the manuscript itself is not clearly presented and often does not discuss the results and conclusions in a systematic manner. The justification for providing a nutritional intervention is not fully stated, and a reader unfamiliar with the subject could be unsure of the biological relationship between TB and nutrition. Overall, the paper could use an additional review by a copyeditor or writer as there are many instances of less-than-clear English, as well as grammatical and punctuation errors (the majority are not listed below due to their prevalence throughout the manuscript).

*1 Line 35: suggest changing “adjuvant” to “adjuvant therapy.”*

Yes, we have changed it to “Adjuvant therapy”

*This text has been corrected.*

*2 Line 47: this sentence states how many people in the intervention/supplement group experienced a favorable outcome. While the p-value is included, the figure for the control group should also be included in the abstract. The definition of “favorable outcome” should also be*

*included here.*

The figure for the control group is included in the abstract and the definition of “favorable outcome” is also included.

*This text has been added and increases clarity for the reader. The results of the study are now much easier to understand when reading the Abstract.*

*3 Lines 51-53: these two sentences seem to repeat each other.*

We have corrected the statement.

*The text has been corrected.*

*4 Lines 65-68: the first sentence of this paragraph describes weight gain during therapy, while the following sentence focuses on malnourishment at time of diagnosis. The two sentences do not logically follow.*

We have modified the paragraph

*These sentences have not been substantially changed, and still do not logically follow.*

*The sentence is changed to have a logical flow.*

*5 Lines 72-74: “multidimensional poor” is not explained, and it is not clear how this connects to undernutrition.*

We have revised the sentence in the background section

*This text has been removed.*

*6 Lines 83-84: “{3, 3, 3, 3, 3, 4}” is not needed.*

As suggested by the reviewer, we have deleted the same.

*This text has been removed.*

*7 Line 85: “Step wedge” should be “stepped wedge” or “stepped-wedge.”*

We have corrected this terminology and made it similar across the manuscript

*This terminology has not been corrected- four different spellings/hyphenations are used in the manuscript.*

*The terminology has been corrected as “Stepped-wedge.”*

*8 Introduction: a more detailed biological explanation of the relationship between nutrition and TB would be helpful.*

A more detailed biological explanation of the relationship between nutrition and TB is added in the introduction

*The biological explanation is not included; i.e.,* ***why*** *undernutrition is associated with poor treatment outcomes for TB.*

*We have tried to include the biological explanation of* ***why*** *undernutrition is associated with poor treatment outcomes for TB in the introduction.*

*9 Introduction: there is useful information included here, but it is not presented in a completely clear manner. It would be helpful to have a more distinct delineation between: a) the relationship between nutrition and TB; b) the results of previous nutritional interventions; and c) the aims of this study.*

We have revised the same to have a more distinct delineation between the relationship between nutrition and TB, the results of previous nutritional interventions and the aims of this study.

*The Introduction is now better organized.*

*10 Methods: a justification for why the stepped-wedge approach was used would be useful here.*

We have added the justification in the methods section.

*The text now states “offered a logical as well as ethical feasibility.” However, a further explanation would be useful.*

*A further justification for why the stepped-wedge approach was used is described below*

*Stepped wedge design (Phased Implementation) is particularly useful when it is not feasible to provide the intervention to every individual/community at once. The intervention is rolled-out sequentially to the trial participants (either as individuals or clusters of individuals) over a number of time periods. The order in which the different individuals or clusters receive the intervention is determined at random and, by the end of the random allocation, all individuals or groups will have received the intervention. This design is for evaluating the effectiveness of interventions that have been shown to be efficacious in a more limited, research setting and are now being scaled up to the community level and also useful for evaluating temporal changes in the intervention effect.*

*11 Line 108: why was the food amount provided sufficient for five people for one month? Was only the patient intended to eat the food, or also their family members (and if so, what about households of different sizes)?*

Family members were not intended, but we provided the same to ensure that the food supplement reaches the patient through this.

*This should be explained within the text.*

*It is now explained within the text.*

*12 Line 113: how was medication adherence assessed?*

Adherence to anti-TB treatment and supplement was ensured by surprise home visits and requisitioning of patient during monthly visits.

*This text has been added and increases clarity. Please change “requisitioning” to “requestioning,” or simply “questioning.”*

*It has been changed.*

*13 Line 133: an explanation of why 74% were male would be helpful here. (Are more TB patients male? Did more men consent to participate compared to women?)*

Yes, 74% were male as we know that TB is more common in males.

*This answer would be helpful within the paper itself.*

*It is included in the paper itself.*

*14 Lines 133-134: with so many having such a low BMI, was the risk of refeeding syndrome addressed?*

Risk of refeeding syndrome was not addressed. Though there were more number of low BMI in the study it was equally distributed in the supplement and no supplement group.

*Please add this to the Limitations, or add text elsewhere in the paper explaining why refeeding syndrome was not addressed.*

*It has been added to the limitations section.*

*15 Lines 136-138 present an aIRR, while lines 144-145 present an aRR for the same outcomes. These figures are nearly identical- is there an error here?*

We have corrected the same.

*The repeated text has been removed.*

*16 Lines 169-171: information on previous nutritional interventions would be helpful in the Introduction, then could be discussed further in the Discussion section.*

The information on the previous nutrition interventions is included in the introduction and discussed in the Discussion section.

*The addition of information on previous nutritional interventions has greatly strengthened the Discussion section.*

*17 Line 179: the psychosocial effects could be discussed at greater length.*

We have addressed the psychosocial effects and have provided the data in detail in Table – 5.

*Psychosocial effects are not defined. There is no Table 5 included- does this refer to Table 4?*

*Psychological and social effects are mentioned in Table 4.*

*18 Lines 184-185: the justification for the stepped-wedge approach presented here is compelling, and should be introduced earlier in the manuscript. Moreover, the comparison at different time points is not discussed within the body of the paper.*

Yes, we have added the same in the methodology section

*More information about the stepped-wedge approach has been added, making the methodology much clearer. However, see comment 10, above.*

*19 Lines 187-188: is this a limitation, or an inherent aspect of using a stepped-wedge approach?*

We do agree that the limitation is an inherent aspect of using a stepped-wedge approach.

*The text included here is now clearer.*

*20 Line 189: the limitations should be discussed at greater length.*

We have added the limitations in the discussion section.

*Additional limitations have not been added; further discussion of limitations would improve the paper.*

*Additional limitations on refeeding syndrome is added.*

*21 Discussion: what are the main lessons learned/recommendations for future interventions?*

We have added the main lessons learned/recommendations for future interventions to the

conclusion.

*Some recommendations for future interventions have been added.*

*22 Table 1 combines the baseline characteristics and the results. The age, gender, baseline BMI, etc. are appropriate for the demographics table (Table 1), but change in weight, etc. would likely be more appropriate in a results table. Moreover, including p-values in Table 1 may not be relevant.*

Table 1 compares the supplement and non-supplement and the p-value is provided to confirm the unbiased and equal allocation in both the groups.

*My preference is to include ‘change in weight’ and ‘change in BMI’ in a results table, rather than in Table 1; however, I acknowledge that including these data in Table 1 is a valid stylistic choice.*

*23 Table 1: did the analysis address factors such as differential alcohol consumption and sputum smear grading between the groups?*

Yes, we have provided the details of the alcohol consumption, and smear grading between the

supplement and non-supplement in Table 1

*These factors are not discussed within the manuscript. The differential alcohol consumption and sputum smear grading between the groups could have affected the results.*

*All potential factors considered for the model in the articles were tested for collinearity with other independent factors and confounders to the outcome or the groups. After this evaluation, only the appropriate factors were included in the analysis. Alcohol consumption and smear grading of the participant are not significantly associated with each other, although they do significantly differ between the groups. These two covariates were considered and adjusted in all the models and findings. We have discussed the same under the statistical consideration section.*

*24 Table 2 and Table 3 would be improved with gridlines.*

Yes, we have added the gridlines as suggested.

*The gridlines have been added.*

*25 Figure 1: the different colors are not explained.*

Yes, we have added a footnote, that the shaded one indicated that the nutrition supplement was rolled out

*The footnote has been added to the figure.*

**Additional comments**

*Lines 53-54: The meaning of the sentence “The changes from the baseline were significantly higher in the supplement group in terms of nutritional status (p<0.001).” could be expressed more clearly.*

Nutritional status was measured in terms weight, waist circumference, BMI and MAC and is discussed in detail in the results section.

*Lines 114-118: This information may be better presented in a table.*

*We have provided the information in a box.*

*Lines 191-194: This sentence is unclear.*

*We have modified the same.*

*Discussion: this section is not structured logically, with information on the role of food supplements, the impact of undernutrition on mortality, etc. presented at different times in the Discussion.*

*Lines 239-241: The text “In this study, more deaths were observed in the supplement group which could be attributed to the fact that most of the participants were extremely underweight in this group” highlights a very important finding/limitation of this study, and should be discussed at greater length. In addition, the following sentence “Thus, early rapid correction of malnutrition with food supplements can prevent deaths due to TB” is not proven by the text that comes before it.*

*We have added more information on the same.*

*The manuscript’s grammar and sentence structure have significantly improved in this revision. However, there are still areas requiring review.*
