# Supplementary material for: Effectiveness of food supplement on treatment outcomes and quality of life in pulmonary tuberculosis: Phased implementation approach
Source: PLoS One. 2024 Jul 16;19(7):e0305855. doi: 10.1371/journal.pone.0305855 (PMC11251611; doi:10.1371/journal.pone.0305855)
Supplement: Attachment — Submitted filename: PONE-D-23-12272 R3 Response to reviewers 29042024.docx [file pone.0305855.s006.docx]

**PONE-D-23-12272 - Response to Reviewers comments (R4)**

1.The authors should strongly consider providing more detailed descriptive statistics regarding the outcomes in each arm. Given the interest in understanding the mechanism by which the nutritional intervention improves outcomes, I would expect that they would have provided a table with the number of participants having 1. Treatment success or cure 2. Death 3. Failure 4. Loss-to-follow up in the nutritional supplementation and the control group. The authors have nicely analyzed differences in outcomes using regression models, but raw numbers in addition would help readers better understand what drove the improved outcomes. This has been done well previously in an earlier study from Bengal (PMID: 28042591)

*Response: Thank you. As suggested, a detailed descriptive statistics regarding the outcomes in each arm is mentioned in the result section lines 181-186 (treatment outcomes) and in detail in the Table 2 (highlighted) in the manuscript.*

2. Given that nutrition is a gendered topic with women routinely having worse nutritional intake than men, it would also be helpful to assess differences in men and women in terms of weight gain and providing that at least as a supplementary figure. There is a paucity of such excellent nutritional supplementation studies in PWTB so these data are very valuable and informative to the community of researchers and scientists. This has been done well in a recent nutritional supplementation study in Madhya Pradesh (<https://doi.org/10.1016/j.cegh.2021.100782>)

*Response: Yes, we also agree that nutrition is a gendered topic with women routinely having worse nutritional intake than men and towards this we did a subgroup analysis to assess the differences in men and women in terms of weight gain. The comparison of weight gain between male and female participants in our study did not reveal any significant differences or associations. Hence, we focused on the differences in weight gain during the second and sixth months, as well as its association with outcomes for each gender separately. In the multivariate analysis we have adjusted for the gender to ensure our findings.*

3. I was unsure why the authors selected the BMI cutoffs that they did. The more traditional

BMI cutoffs, as defined by the WHO, are BMI <16 kg/m2 for severe undernutrition and 16-18.5 would be considered mild-moderate. 18.5-24.9 is considered “normal,” as the authors have suggested, but a BMI >23 is generally considered overweight among South Asians. I recommend adjusting BMI values to more conventional thresholds to facilitate comparability with large nutrition-TB studies such as the recent RePORT India publication (PMID: 36424864). If not, please provide a justification.  
*Response: Thank you, for the comment. We had taken the reference from Guidance Document - Nutritional Care & Support for TB patients in India 2017.We have modified the table 1 and also the reference in the manuscript.*

4. Please state more clearly how long your intervention was. I inferred it was just 1 month long from the text, but this should be stated very clearly. This is critical to allow comparison. In general, I think the presentation of the nutritional intervention could be perhaps improved. I recommend adapting the table to resemble the informative table 1 from this protocol paper for an ongoing nutritional intervention (PMID: 34641820),

*Response:* *Thank you for the suggestion. The presentation of the nutritional intervention is*

*modified in the box below. Also, it is revised in the manuscript. Also, it is revised in the*

*manuscript.*

| Food category | Food item | Quantity (in kgs)  RDA | Energy (Kcals) | Protein  (g) | Fat  (g) | Carbohydrates  (g) | Others |
| --- | --- | --- | --- | --- | --- | --- | --- |
| Grains | **Rice** | 100/day | 162 | 3.17 | 1.28 | 33.17 | Vitamin D - 0  Calcium 12mg -1%  Iron-1.42mg-8%  Potassium-42mg-1%  Vitamin A-10mcg 1%  Dietary fibre 67g |
|  | **Ragi** (Millet) | 100gm | 344 | 7.3g | 1.3g | 72.0g | Folic Acid- 18.3ug  Carotene – 42ug |
| Pulse | Local Arhar dhal (Kandol) | 40.0 | 360 | 20.8 | 5.6 | 59.8 | Calcium -56mg  Phosphorus- 331mg  Iron – 5.3mg |
| Fat | Mustard Oil | 20 | 900 | -- | 100 | -- | -- |
|  | Sathu* | 50gms | 413.3 | 19 | 6 | 71 | Vit A 300IU,  Vit C 300mg Vit D 100 IU  Calcium 200mg |

*RUCHI Brand (local) - Flour made from groundnut, wheat, flat rice, and chickpea

Ref: C.Gopalan et.al, Nutritive value of Indian Foods, ICMR- NIN.

5. Please comment on the socioeconomic status of the participants of this study and if possible, include a deprivation measure such as the kuppuswamy scale (PMC6618222) or at least income in your analysis if possible. Poverty is a driver of unfavorable outcomes and can be an unmeasured confounder. If it is not possible to include it in this analysis, at least describe the general economic status of the population (e.g. is it comparable to the extremely impoverished population of the RATIONS study?) and acknowledge this as a study limitation.
*Response: Yes, we also agree that poverty is a driver of unfavorable outcomes and can be an unmeasured confounder.But,the comparison of income between the groups did not show any significant differences or associations either within the groups or across the outcomes.(Table 1)*

Minor points:

 1. Please use person-first language. Instead of TB patient, please use person with TB

(PWTB) as that is less stigmatizing

*Response: We have revised the sentence in the text (highlighted).*

2. Sentence seems incomplete: “…500gms of “Sathu” (flour made from 112 groundnut, wheat,

flat rice, and chickpea which contained the following - protein 19gm, fat 113 6gms,

carbohydrates 71gm, calcium 200mg, energy 413.31kcal, Vit A 300IU, Vit C 300mg 114 and

Vitamin D100IU.”
 *Response: We have revised the sentence in the text (highlighted).*

3. I was surprised to see two of the three largest TB outcome studies from India excluded from

the paper’s discussion in favor of smaller studies from non-Indian populations:

(PMID: 24205052 and PMID: 36424864)

*Response: We have included PMID 24205052 in our discussion (reference no.11) (highlighted). PMID 36424864 was published after we submitted the manuscript to the journal. Hence, we were not able to reference the paper in our manuscript.*
