# Supplementary material for: Effectiveness of food supplement on treatment outcomes and quality of life in pulmonary tuberculosis: Phased implementation approach
Source: PLoS One. 2024 Jul 16;19(7):e0305855. doi: 10.1371/journal.pone.0305855 (PMC11251611; doi:10.1371/journal.pone.0305855)
Supplement: Attachment — Submitted filename: PONE-D-23-12272 R4 Response to reviewers 27052024.docx [file pone.0305855.s007.docx]

**PONE-D-23-12272 - Response to Reviewers comments (R5)**

Reviewer #4

1. After reading the revised manuscript, I am still confused what the duration of nutritional support was: "The total quantity provided supported a family of five for one month and cost Rs.1500/- per month." Does this mean that that they received the supplementation just for one month or throughout the treatment at a cost of 1500 rupees per month.

Please state specifically that the supplementation was provided for x months.

*Response: Thank you for the comment. The food supplement was supplied to the index patient as fortnightly packs at the Directly observed treatment short course (DOTS) Center by the study staff till the end of his /her treatment period. We have now stated this specifically in the revised version of the manuscript. (Line 117-119 -highlighted)*

2. As the authors know, income is an imperfect marker of socioeconomic status, but it is better than nothing. In their response to the reviewer, they stated no differences between the control and the intervention group in income, but I don't see this in their descriptive statistics table. This should be added. And if there is a significant difference, it should be included in the multivariable model.

*Response: Thank you, we also agree to the comment and we have added the same in the descriptive statistics table. (Table 1-highlighted)*
