# Supplementary material for: Effectiveness of food supplement on treatment outcomes and quality of life in pulmonary tuberculosis: Phased implementation approach
Source: PLoS One. 2024 Jul 16;19(7):e0305855. doi: 10.1371/journal.pone.0305855 (PMC11251611; doi:10.1371/journal.pone.0305855)
Supplement: Attachment — Submitted filename: Response to the Reviewer comments.docx [file pone.0305855.s002.docx]

Response to Reviewer’s Comments: **Effectiveness of Food Supplement on Treatment Outcomes and Quality of Life in Pulmonary Tuberculosis: Phased Implementation Approach**

|  | | |
| --- | --- | --- |
| S. No. | Comments | Response |
| Reviewer #1: The study entitled “Effectiveness of Food Supplementation on Treatment Outcomes and Quality of Life in Pulmonary Tuberculosis: Phased Implementation Approach” evaluated the influence of early food supplementation on the TB treatment outcome and quality of life. The following are the suggestions that the authors may consider adding: | | |
| 1 | The discussion is minimal with only 3 references. Several RCTs and other study types have been published with respect to nutritional/food supplementation in TB patients in India and elsewhere. The authors may discuss this with respect to these studies. | We have added more references and revised the discussion accordingly |
| 2 | The authors have mentioned that food supplementation with rice, finger millet, kandul dal, mustard oil, and sathu was supplied. What is the protein, carbohydrate, minerals, etc. available per gram in these foods? The discussion on this is important since it gives an idea to readers regarding the various macro and micronutrients that were supplied to the patients. | This has been now added to the manuscript |
| 3 | The authors may discuss the various nutritional deficiencies linked to the risk of developing TB and how the current food supplementation could aim in correcting nutritional deficiencies that could be related to improving the TB outcomes such as smear-negative/culture-negative status etc. | We have revised this in the background and discussion section. |
| 4 | Since it is mentioned in the introduction that “It is encouraging to note studies showing TB patients who received food supplements during their initial phase of ATT gained weight, had a shorter time to sputum conversion….”, it would be interesting to add information on the influence of food supplementation and an increase in BMI on sputum smear grading in results and discussion. | We have added information on the influence of food supplementation and an increase in BMI on sputum smear grading in the results and discussion. |
| 5 | What is the average cost incurred for providing food supplementation in the quantities mentioned per family? This may be important for budget planning for programmatic implementation. | The cost of the food supplement was budgeted as Rs.1500/- per family. However, we have not done a cost-effectiveness analysis as part of this study. |
| 6 | The authors should add more information pertaining to the results and discussion regarding the impact of food supplementation on HRQOL and lung health. | We have added more information on the impact of food supplementation on HRQOL and lung health in the results and discussion section. |
| 7 | Typo errors such as of “Hyper Tension” (table 1) can be corrected. | We have corrected this in Table 1 |
| Reviewer #2: The topic of providing a nutritional intervention for persons with TB is important, and the results present compelling evidence for the benefits of such an intervention. However, the manuscript itself is not clearly presented and often does not discuss the results and conclusions in a systematic manner. The justification for providing a nutritional intervention is not fully stated, and a reader unfamiliar with the subject could be unsure of the biological relationship between TB and nutrition. Overall, the paper could use an additional review by a copyeditor or writer as there are many instances of less-than-clear English, as well as grammatical and punctuation errors (the majority are not listed below due to their prevalence throughout the manuscript). | | |
| 1 | Line 35: suggest changing “adjuvant” to “adjuvant therapy.” | Yes, we have changed it to “Adjuvant therapy” |
| 2 | Line 47: this sentence states how many people in the intervention/supplement group experienced a favorable outcome. While the p-value is included, the figure for the control group should also be included in the abstract. The definition of “favorable outcome” should also be included here. | The figure for the control group is included in the abstract and the definition of “favorable outcome” is also included. |
| 3 | Lines 51-53: these two sentences seem to repeat each other. | We have corrected the statement. |
| 4 | Lines 65-68: the first sentence of this paragraph describes weight gain during therapy, while the following sentence focuses on malnourishment at time of diagnosis. The two sentences do not logically follow. | We have modified the paragraph |
| 5 | Lines 72-74: “multidimensional poor” is not explained, and it is not clear how this connects to undernutrition. | We have revised the sentence in the background section |
| 6 | Lines 83-84: “{3, 3, 3, 3, 3, 4}” is not needed. | As suggested by the reviewer, we have deleted the same. |
| 7 | Line 85: “Step wedge” should be “stepped wedge” or “stepped-wedge.” | We have corrected this terminology and made it similar across the manuscript |
| 8 | Introduction: a more detailed biological explanation of the relationship between nutrition and TB would be helpful. | A more detailed biological explanation of the relationship between nutrition and TB is added in the introduction |
| 9 | Introduction: there is useful information included here, but it is not presented in a completely clear manner. It would be helpful to have a more distinct delineation between: a) the relationship between nutrition and TB; b) the results of previous nutritional interventions; and c) the aims of this study. | We have revised the same to have a more distinct delineation between the relationship between nutrition and TB, the results of previous nutritional interventions and the aims of this study. |
| 10 | Methods: a justification for why the stepped-wedge approach was used would be useful here. | We have added the justification in the methods section. |
| 11 | Line 108: why was the food amount provided sufficient for five people for one month? Was only the patient intended to eat the food, or also their family members (and if so, what about households of different sizes)? | Family members were not intended, but we provided the same to ensure that the food supplement reaches the patient through this. |
| 12 | Line 113: how was medication adherence assessed? | Adherence to anti-TB treatment and supplement was ensured by surprise home visits and requisitioning of patient  during monthly visits. |
| 13 | Line 133: an explanation of why 74% were male would be helpful here. (Are more TB patients male? Did more men consent to participate compared to women?) | Yes, 74% were male as we know that TB is more common in males. |
| 14 | Lines 133-134: with so many having such a low BMI, was the risk of refeeding syndrome addressed? | Risk of refeeding syndrome was not addressed. Though there were more number of low BMI in the study it was equally distributed in the supplement and non-supplement group |
| 15 | Lines 136-138 present an aIRR, while lines 144-145 present an aRR for the same outcomes. These figures are nearly identical- is there an error here? | We have corrected the same. |
| 16 | Lines 169-171: information on previous nutritional interventions would be helpful in the Introduction, then could be discussed further in the Discussion section. | The information on the previous nutrition interventions is included in the introduction and discussed in the Discussion section |
| 17 | Line 179: the psychosocial effects could be discussed at greater length. | We have addressed the psychosocial effects and have provided the data in detail in Table – 5. |
| 18 | Lines 184-185: the justification for the stepped-wedge approach presented here is compelling, and should be introduced earlier in the manuscript. Moreover, the comparison at different time points is not discussed within the body of the paper. | Yes, we have added the same in the methodology section |
| 19 | Lines 187-188: is this a limitation, or an inherent aspect of using a stepped-wedge approach? | We do agree that the limitation is an inherent aspect of using a stepped-wedge approach. |
| 20 | Line 189: the limitations should be discussed at greater length. | We have added the limitations in the discussion section. |
| 21 | Discussion: what are the main lessons learned/recommendations for future interventions? | We have added the main lessons learned/recommendations for future interventions to the conclusion. |
| 22 | Table 1 combines the baseline characteristics and the results. The age, gender, baseline BMI, etc. are appropriate for the demographics table (Table 1), but change in weight, etc. would likely be more appropriate in a results table. Moreover, including p-values in Table 1 may not be relevant. | Table 1 compares the supplement and non-supplement and the p-value is provided to confirm the unbiased and equal allocation in both the groups. |
| 23 | Table 1: did the analysis address factors such as differential alcohol consumption and sputum smear grading between the groups? | Yes, we have provided the details of the alcohol consumption, and smear grading between the supplement and non-supplement in Table 1 |
| 24 | Table 2 and Table 3 would be improved with gridlines. | Yes, we have added the gridlines as suggested. |
| 25 | Figure 1: the different colors are not explained. | Yes, we have added a footnote, that the shaded one indicated that the nutrition supplement was rolled out |
| Reviewer #3: Thank you for the opportunity to review this manuscript investigating the effectiveness of food supplementation on treatment outcomes in patients with TB. Remaining one of the leading infectious causes of morbidity and mortality the research is both timely and relevant to expand the evidence base re supplementary feeding in this context. It was good to note that the researchers also investigated aspects of QoL, which has been lacking in other studies.  Please consider the comments/request for clarity below: | | |
| 1 | Abstract repeat - please correct? Improvement in nutritional status was observed in the supplement group (p<0.001). The supplement group experienced an improvement in nutritional status (p 0.001). Clarity is needed re the phased approach: rationale for this approach? It was difficult to deduct how the process unfolded, despite the inclusion of Figure 1. The figure gives the impression that Cluster 1 received the supplement for an extended period of time and longer than the other clusters. It is stated that the last cluster only received the supplement for 2 months? Please clarify for the reader, also in the article itself. | We have revised the abstract.  In stepped wedge design, the clusters will have intervention periods that vary across and the same has been shown in figure – 1. |
| 2 | Please provide more information regarding the food supplement (calories, protein, and other details). Was the idea that the supplement is for use by the whole family? "Food supplement could feed a family of 5 for a month", but provided every 2 weeks? Did the researchers check/ask whether the index patient consumed some of the supplements? More details will provide clarity. | We have revised and updated the details. Adherence to anti-TB treatment and supplements was ensured by surprise home visits and requestioning of patients during monthly visits |
| 3 | Include references for the 2 questionnaires included in the study and perhaps brief details re the questionnaires. | We have added the references in the results and the reference section. |
| 4 | Sample: unclear which patients (n=147) did not receive the supplement, and why? The authors could include participant numbers per cluster (those with supplement and those without) to help the reader understand how the study unfolded. The uneven numbers (supplement vs no supplement) are a limitation, but the authors could have realized this at the study design phase. Was it planned as such? Did the researchers employ consecutive sampling? Was there specific inclusion and exclusion criteria? | The distribution of participants enrolled in each cluster and the number under the supplement and non-supplement were clearly shown in Figure – 1b |
| 5 | Results: why did the researchers only report weight and BMI change at 2 months? Longer time-point data available? | The two-month BMI Change is considered the surrogate marker for the TB treatment response. |
| 6 | Discussion: the researchers state the study emphasizes the effect of diet on improving treatment adherence and leading to treatment completion and better outcomes, although not reporting any treatment adherence data under results. How was this measured/evaluated? The discussion is very limited and lacks a critical interrogation of the findings. The authors should also emphasize the novel components of their study and what it adds to the evidence base. | We have used the stepped wedge design in a phased manner, as a family-centric approach. The study was done in a resource-constrained area under a programmatic setting.  We have updated the same in the discussion section. |
| 7 | Abbreviations should be declared for all tables/figures, even if obvious (e.g. LTFUP). | We have declared the abbreviation in the table footnotes for all tables/figures. |
| 8 | The quality of the figures must be drastically improved. | We have revised the quality of the figure using the Plos tool |
