# Supplementary material for: Effectiveness of food supplement on treatment outcomes and quality of life in pulmonary tuberculosis: Phased implementation approach
Source: PLoS One. 2024 Jul 16;19(7):e0305855. doi: 10.1371/journal.pone.0305855 (PMC11251611; doi:10.1371/journal.pone.0305855)
Supplement: Attachment — Submitted filename: Response to the Reviewer comments.docx [file pone.0305855.s004.docx]

Response to Reviewer’s Comments: **Effectiveness of Food Supplement on Treatment Outcomes and Quality of Life in Pulmonary Tuberculosis: Phased Implementation Approach (**PONE-D-23-12272R1)

|  | | |
| --- | --- | --- |
| S. No. | Comments | Response |
| **Reviewer #2**: The topic of providing a nutritional intervention for persons with TB is important, and the results present compelling evidence for the benefits of such an intervention. However, the manuscript itself is not clearly presented and often does not discuss the results and conclusions in a systematic manner. The justification for providing a nutritional intervention is not fully stated, and a reader unfamiliar with the subject could be unsure of the biological relationship between TB and nutrition. Overall, the paper could use an additional review by a copyeditor or writer as there are many instances of less-than-clear English, as well as grammatical and punctuation errors (the majority are not listed below due to their prevalence throughout the manuscript). | | |
| 1 | Lines 65-68: the first sentence of this paragraph describes weight gain during therapy, while the following sentence focuses on malnourishment at time of diagnosis. The two sentences do not logically follow.  *These sentences have not been substantially changed, and still do not logically follow.* | *We have further modified the paragraph and rearranged the sentences so that they flow logically* |
| 2 | Line 85: “Step wedge” should be “stepped wedge” or “stepped-wedge.” *This terminology has not been corrected- four different spellings/hyphenations are used in the manuscript.* | *We apologize for this. We have now corrected this terminology throughout the manuscript* |
| 3 | Introduction: a more detailed biological explanation of the relationship between nutrition and TB would be helpful. *The biological explanation is not included; i.e., why undernutrition is associated with poor treatment outcomes for TB.* | *A biological explanation of the relationship between*  *nutrition and TB is now added in the introduction* |
| 4 | Methods: a justification for why the stepped-wedge approach was used would be useful here. The text now states “offered a logical as well as ethical feasibility.” However, a further explanation would be useful. | We have added the justification in the ‘Methods’ section as well as given it here for using this methodology for this study.  *“**Stepped wedge design (Phased Implementation) is particularly useful when it is not feasible to provide the intervention to every individual/community at once. This design is for evaluating the effectiveness of interventions that have been shown to be efficacious in a more limited, research setting and are now being scaled up to the community level and also useful for evaluating temporal changes in the intervention effect. The intervention is rolled-out sequentially to the trial participants (either as individuals or clusters of individuals) over a number of time periods. The order in which the different individuals or clusters receive the intervention is determined at random and, by the end of the random allocation, all individuals or groups will have received the intervention”* |
| 5 | Line 108: why was the food amount provided sufficient for five people for one month? Was only the patient intended to eat the food, or also their family members (and if so, what about households of different sizes)? This should be explained within the text. | *Food supplement was not intended for the Family members, but we provided the same to all family members hoping ensure that the food supplement reaches the patient and not become insufficient* |
| 6 | Line 113: how was medication adherence assessed?  *Please change “requisitioning” to “requestioning,” or simply “questioning.”* | *The spelling has been corrected* |
| 7 | Line 133: an explanation of why 74% were male would be helpful here. (Are more TB patients male? Did more men consent to participate compared to women?) This answer would be helpful within the paper itself. | Yes, 74% were male as we know that TB is more common in males and *this is now added in the manuscript.* |
| 8 | Lines 133-134: with so many having such a low BMI, was the risk of refeeding syndrome addressed? Please add this to the Limitations, or add text elsewhere in the paper explaining why refeeding syndrome was not addressed. | *Risk of refeeding syndrome was not addressed. This has now been added as a limitation of the study.* |
| 9 | Line 179: the psychosocial effects could be discussed at greater length. | *Psychological and social effects are mentioned in Table 4 nd discussed in Discussion section.* |
| 10 | Lines 184-185: the justification for the stepped-wedge approach presented here is compelling, and should be introduced earlier in the manuscript. Moreover, the comparison at different time points is not discussed within the body of the paper. | *Yes, we have added the same in the methodology section* |
| 11 | Line 189: the limitations should be discussed at greater length. | *We have added the limitations in the discussion section.* |
| 12 | Table 1: The differential alcohol consumption and sputum smear grading between the groups could have affected the results. | *We have now added differential alcohol consumption and smear grading status to the text now*  *All potential factors considered for the model in the articles were tested for collinearity with other independent factors and confounders to the outcome or the groups. After this evaluation, only the appropriate factors were included in the analysis. Alcohol consumption and smear grading of the participant are not significantly associated with each other, although they do significantly differ between the groups. These two covariates were considered and adjusted in all the models and findings. We have discussed the same under the statistical consideration section.* |
| 13 | Lines 53-54: The meaning of the sentence “The changes from the baseline were significantly higher in the supplement group in terms of nutritional status (p<0.001).” could be expressed more clearly. | Nutritional status was measured in terms weight, waist circumference, BMI and MAC and is discussed in detail in the results section. |
| 14 | The text “In this study, more deaths were observed in the supplement group which could be attributed to the fact that most of the participants were extremely underweight in this group” highlights a very important finding/limitation of this study, and should be discussed at greater length. | We have added more information on the same in the discussion section now. |
| 15 | Lines 239-241: The text “In this study, more deaths were observed in the supplement group which could be attributed to the fact that most of the participants were extremely underweight in this group” highlights a very important finding/limitation of this study, and should be discussed at greater length. In addition, the following sentence “Thus, early rapid correction of malnutrition with food supplements can prevent deaths due to TB” is not proven by the text that comes before it. | We have now corrected and added more information on the same. |
